# Personality disorder in an Early Intervention Psychosis cohort: Findings from the Social Epidemiology of Psychoses in East Anglia (SEPEA) study

**DOI:** 10.1371/journal.pone.0234047

**Published:** 2020-06-05

**Authors:** Ka-Young Ban, David P. J. Osborn, Yasir Hameed, Santvana Pandey, Jesus Perez, Peter B. Jones, James B. Kirkbride

**Affiliations:** 1 Division of Psychiatry, UCL, London, United Kingdom; 2 Camden and Islington NHS Foundation Trust, London, United Kingdom; 3 Norfolk & Suffolk Foundation Trust, Norwich, Norfolk, United Kingdom; 4 Department of Psychiatry, University of Cambridge, Cambridge, United Kingdom; 5 Cambridgeshire & Peterborough Foundation Trust, and NIHR Collaboration for Leadership in Applied Health Research and Care (CLAHRC) East of England, Cambridge, Cambridgeshire, United Kingdom; Department of Psychiatry and Neuropsychology, Maastricht University Medical Center, NETHERLANDS

## Abstract

**Aim:**

Personality Disorders (PD) often share clinical and phenomenological overlap with psychotic disorders, especially at onset. However, there is little research on comorbid PD among people experiencing first episode psychosis. We examined the prevalence of PD recording and its sociodemographic and clinical correlates in people accepted to Early Intervention in Psychosis (EIP) services.

**Methods:**

Participants were aged 16–35, accepted into 6 EIP services for suspected psychosis, as part of the Social Epidemiology of Psychoses in East Anglia (SEPEA) study. PD was recorded by clinicians according to ICD-10. Multilevel logistic regression was performed.

**Results:**

Of 798 participants, 76 people (9.5%) received a clinical diagnosis of PD, with emotionally unstable PD (75.0%, N = 57) the most common subtype. In multivariable analysis, risk factors for PD included female sex (odds ratio [OR]: 3.4; 95% CI: 2.0–5.7), absence of psychotic disorder after acceptance to EIP (OR: 3.0; 95% CI: 1.6–5.5), more severe hallucinations (OR: 1.6; 95% CI: 1.2–2.1), and lower parental SES (OR: 1.4; 95% CI: 1.1–1.8). Compared with the white British, black and minority ethnic groups were less likely to receive a PD diagnosis (OR: 0.3; 95% CI: 0.1–0.7). There was no association between PD and neighbourhood-level deprivation or population-density.

**Conclusions:**

Recording of a PD diagnosis was three times more common amongst participants later found not to meet threshold criteria for psychotic disorder, implying phenomenological overlap at referral which highlights difficulties encountered in accurate diagnostic assessment, treatment and onward referral. People with PD experienced more individual-level, but not neighbourhood-level social disadvantage in an already disadvantaged sample.

## Introduction

People with personality disorders (PD) are thought to be at high risk of other psychiatric disorders including mood, anxiety and substance use disorders [1,2]. Although there is no consolidated consensus on the prevalence of comorbid PD and psychotic disorders, a systematic review suggested this could be as high as 39.5% (95% CI 25.2%-55.8%), though this varied substantially between studies, possibly due to variation by country, study type or the diagnostic tools used to assess and record PD [3]. In a recent study, more than one third of people with borderline PD reported comorbid psychotic symptoms [4]. Further, in the National Epidemiologic Survey on Alcohol and Related Conditions (NESARC), people with schizophrenia and other psychotic disorders showed high rates of various forms of co-occurring PD [5]. PD subtypes associated with psychotic disorders in this study included paranoid, schizoid, antisocial, histrionic, avoidant, dependent and obsessive-compulsive PD.

Both PD and psychotic disorder are known to start developing at an early stage in life [6,7]. Young people with problems in personality functioning exhibit symptoms similar to those of psychosis such as hallucinations, potentially making the conditions difficult to distinguish at first presentation [8]. Comorbid PD is associated with worse outcomes, such as increased relapse and readmission to hospital as well as more violent behaviours in people with psychosis [9,10]. Existing studies which have estimated the prevalence of PD in first episode psychosis (FEP) samples in EIP care have largely been based on small samples, which may have led to imprecise estimates of comorbidity. For example, in a study on forty-nine patients using an EIP service, the prevalence of comorbid PD was estimated to be 45% [11], while a further study estimated that 50% of a sample of fifty-five people with FEP met criteria for two or more PDs [12]. Large, population-based epidemiological data on this issue remains absent.

Furthermore, little is known about possible predictors of comorbid PD in FEP samples, which may be helpful in informing both clinical practice and aetiology. At the individual level, PD and psychosis may share phenomenological overlap, including a relatively early onset and psychotic symptoms including hallucinations, particularly in people with borderline PD [13–15], although these tend to be more transient than in people with psychotic disorders. Both PD and psychotic disorders can also present with affective symptoms including emotional disturbances and self-harm [16–20], and may share sociodemographic correlates including single relationship status and lower socioeconomic status [21–23]. Interestingly, PD prevalence appears to be higher for men in community settings, but higher for women in clinical settings [24,25]. People of white ethnicity have higher prevalence than ethnic minority groups, in contrast to what is typically observed in FEP [26,27]. Both PD and FEP appear to be more common in deprived neighbourhoods [26,28], though no epidemiological studies have investigated whether social adversity is associated with comorbid PD in people with FEP.

Previous studies on the prevalence of PD among FEP samples in EIP services have relied on data obtained for research purposes, and there has been no attempt to explore whether prevalence of PD shows consistent results when using PD data collected prospectively as part of EIP services routine clinical practice. Hence, in the present study, we sought to estimate the prevalence of PD documented in a large, naturalistic cohort of people accepted for care in EIP services for FEP. We investigated whether sociodemographic and clinical characteristics differed between people with and without PD diagnoses in this context, including markers of individual and neighbourhood-level social disadvantage. We hypothesized that PD prevalence would increase with younger age at first contact, female sex, white British ethnicity, single/divorced marital status, lower socioeconomic status (SES), and greater neighbourhood deprivation and population density.

## Materials and methods

### Design and setting

We obtained data from the Social Epidemiology of Psychoses in East Anglia (SEPEA) study, originally designed to investigate variation in the incidence of psychotic disorders in rural England [26]. Case ascertainment took place between 1 August 2009 and 31 January 2013 in Cambridgeshire and Peterborough NHS Foundation Trust (CPFT), and from 28 September 2009 to 28 March 2013 in the Norfolk and Suffolk NHS Foundation Trust (NSFT).

### Inclusion criteria and participants

Participants accepted into EIP services at first referral for suspected psychosis were included in the present study if they met the following inclusion criteria:

1. Acceptance to EIP services
2. Aged between 16–35 in NSFT, or 17–35 in CPFT
3. Resident in the defined catchment area
4. No previous contacts with mental health services for psychotic symptoms, or previous treatment involving antipsychotic medication for more than 6 months.

### Procedures

We collected sociodemographic data at baseline. Clinical information was collected at two time points: 6 months after acceptance into EIP care and at discharge from EIP services (up to 3 years of care); at these time points the clinicians responsible for care provided primary and secondary International Classification of Diseases, Tenth Revision (ICD-10) clinical diagnoses.

### Outcome variable

In the present paper, our main outcome was a clinical diagnosis of ICD-10 PD (F60.X) as a primary or secondary diagnosis at either time point during EIP care (6 months post-acceptance, or discharge).

### Exposure variables

We considered FEP (yes/no) as a predictor of PD. We defined FEP as a clinical diagnosis of an ICD-10 psychotic disorder (F10-33) at either time point, subsequently confirmed by a standardised research-based diagnosis using the OPCRIT assessment. OPCRIT is known to produce reliable and valid ICD-10 diagnoses for major psychotic and affective disorders based on rating of 90 signs and symptoms of disorder [29,30]. A panel of trained diagnosticians conducted OPCRIT assessments, with acceptable inter-rater reliability as previously reported [26].

We obtained data on age (continuous), sex (male/female), ethnicity, marital status, individual and parental SES and country of birth at EIP acceptance. Age was defined as age-at-first-referral to EIP care. Ethnicity was self-ascribed to one of 18 categories in the 2011 census, recoded here as a binary variable (white British versus black and minority ethnic (BME) groups). Marital status was classified as married/civil partnership, divorced/dissolved/ separated or single. Occupational data on participants and their parents was classified into five (participants) or four (parents) categories according to the National Statistics Socio-Economic Classification guidelines[31], as professional/managerial, intermediate occupations, routine/manual, and people not in employment, with students in a fifth category for participant SES.

We geo-coded participants to their small area of residence using postcode information for neighbourhood-level exposures on PD risk. We used “electoral wards” as our neighbourhood-level of analysis, with 530 wards in the SEPEA catchment area, with a median population of 3,992 people (interquartile range (IQR): 2,426–5,935). Population density was estimated based on the 2011 census population divided by area size in hectares (people per hectare (ppha)), and categorized into four equal-interval groups (0–4,000; 4,001–8,000; 8,001–12,000; and over 12,000 ppha). Multiple deprivation was defined as the proportion of households in each neighbourhood categorized as deprived on two or more of four indicators from the 2011 census covering employment, education, health, and living environment (Kirkbride et al., 2017), with four equal-interval scale categories (7.7%-18%, 18.1%-28%, 28.1%-38%, and 38.1%-47.1%).

We included waiting time (in weeks) between referral and acceptance by EIP services [32] and symptom dimensionality as potential predictors of PD in our analyses. Symptom dimensions were derived from a factor analysis of OPCRIT items, as previously described [33], for which we included scores on seven dimensions: mania, depressive symptoms, other delusions, psychomotor poverty and disorganisation, first rank delusions, paranoia and hallucinations.

### Statistical analysis

We used Chi-square (χ2) tests or Fisher’s exact test (FET), and independent t-tests or Wilcoxon Rank sum-tests (RST), as appropriate, to investigate univariable differences in PD prevalence by sociodemographic and clinical characteristics. We then conducted multilevel logistic regression to examine the joint effects of these factors on PD prevalence. We used Akaike’s Information Criterion (AIC) to determine entry order into a multivariable model, with lower AIC scores indicating better model fit, and employed a forward selection method to identify the best-fitting set of predictors associated with PD. We used likelihood ratio tests (LRT) to determine the best-fitting model, with statistical significance set at p<0.05. We conducted complete case analysis due to minimal missing data (N = 39, 4.9%). Analyses were conducted in Stata version 14.

### Ethics

Ethical approval was granted by the Cambridgeshire III Local Research Ethics Committee (09/H0309/39). All data have been fully anonymized before we accessed them for analysis, and we also acquired written consent from the patient for using their medical records to be used in research.

## Results

### Demographic and clinical characteristics

We identified 798 participants who met inclusion criteria, of whom 687 (86.1%) received a research-based diagnosis of FEP and 76 (9.5%) received a clinical PD diagnosis (Table 1). Fifty-two of those diagnosed with FEP (7.5%) received a comorbid PD diagnosis. The median age-at-first referral to EIP of people with and without any recorded PD was 20.5 (IQR: 18.2–23.9) and 22.6 (IQR: 19.4–27.2) years old, respectively (RST: 3.4; p<0.01). There were slightly more women (57.9%, N = 44) than men (42.1%, N = 32) with PD, whereas two thirds (67.7%, N = 489) of people without PD were men (χ2 test: 19.9; p<0.01). Participants with PD were also more likely to be white British (FET; p<0.01), from low SES (individual SES: FET, p = 0.04; parental SES: χ2 test: 11.1, p = 0.01), UK-born (FET; p<0.01), and were less likely to be diagnosed with FEP (χ2 test: 21.9; p<0.01); they also had longer median waiting times between referral and acceptance to EIP (RST: -2.0; p = 0.04). Participants with PD differed on most psychopathology dimensions at initial assessment, and were rated as having more hallucinations, paranoia and depressive symptoms than those without PD (Table 1), but fewer manic, negative and first rank delusional symptoms. There were no significant differences between participants with and without PD in neighbourhood-level population density (χ2 test: 2.4; p = 0.48) or multiple deprivation (χ2 test: 3.4; p = 0.34).

**Table 1:** Demographic and clinical characteristics of people within EIP service.

	PD diagnosis (n = 76)		No PD diagnosis (n = 722)		statistic	
	n	(%) or (IQR^¶^)	n	(%) or (IQR)	*test*	p
Age (years)					3.4†	<0.01
Median (IQR)	20.5	(18.2–23.9)	22.6	(19.4–27.2)		
Sex					19.9	<0.01
Female	44	(57.9)	233	(32.3)		
Male	32	(42.1)	489	(67.7)		
Ethnicity					‡	<0.01
White British	71	(93.4)	542	(75.1)		
BME	5	(6.6)	180	(25.0)		
Marital Status					‡	0.67
Married/Civil partnership	5	(6.6)	67	(9.4)		
Single	69	(90.8)	633	(88.7)		
Divorced/Dissolved/Separated	2	(2.6)	14	(2.0)		
Missing data	0		8			
Participant SES					‡	0.04
Professional & managerial	3	(4.0)	75	(10.4)		
Intermediate	4	(5.3)	87	(12.1)		
Routine	33	(43.4)	278	(38.5)		
Student	15	(19.7)	157	(21.8)		
LR unemployed or NW	21	(27.6)	125	(17.3)		
Parental SES					11.1	0.01
Professional & managerial	10	(13.2)	222	(30.8)		
Intermediate	18	(23.7)	156	(21.6)		
Routine & manual	25	(32.9)	192	(26.6)		
Long-term unemployed, not working or student	23	(30.3)	152	(21.1)		
Country of birth					‡	<0.01
Born in UK	74	(97.4)	610	(84.5)		
Foreign born	2	(2.6)	112	(15.5)		
First Episode Psychosis (FEP)					21.9	<0.01
Yes	52	(68.4)	635	(88.0)		
No	24	(31.6)	87	(12.1)		
Waiting time (weeks)					-2.0†	0.04
Median (IQR)	2.29	(1.36–5.86)	2.14	(1–4.14)		
Symptoms of Psychosis (Median & IQR)						
Mania	-0.31	(-0.72–0.37)	-0.15	(-0.57–0.58)	1.9†	0.05
Depressive symptoms	0.52	(-0.45–1.15)	-0.05	(-0.84–0.70)	-3.4†	<0.01
Other delusions	-0.25	(-0.66–0.58)	-0.05	(-0.63–0.76)	0.8§	0.41
Psychomotor poverty & disorganisation	-0.29	(-0.89–0.31)	-0.01	(-0.54–0.63)	2.6†	<0.01
First rank delusions	-0.23	(-0.77–0.35)	-0.04	(-0.53–0.59)	2.3†	0.02
Paranoia	0.18	(-0.46–0.88)	-0.07	(-0.72–0.64)	-2.1§	0.04
Hallucinations	0.45	(-0.24–0.99)	-0.11	(-0.73–0.64)	-3.9§	<0.01
Population Density					2.4	0.48
0–4,000 ppha	29	(40.9)	315	(45.3)		
4,001–8,000 ppha	16	(22.5)	135	(19.4)		
8,001–12,000 ppha	18	(25.4)	137	(19.7)		
12,001-max ppha	8	(11.3)	109	(15.7)		
Missing data	5		26			
Multiple Deprivation					3.4	0.34
7.8–18%	16	(22.5)	172	(24.7)		
18.1–28%	26	(36.6)	312	(44.8)		
28.1–38%	22	(31.0)	164	(23.6)		
38.1–47.1%	7	(9.9)	48	(6.9)		
Missing data	5		26			

†: The Wilcoxon Rank-sum test;

‡: Fisher’s exact test;

§: The independent t-test;

¶: Interquartile range (IQR); PD: personality disorder; BME: black and minority ethnic; PPHA: people per hectare; SES: socioeconomic status

### Sub-types of PD

**Fig 1:**
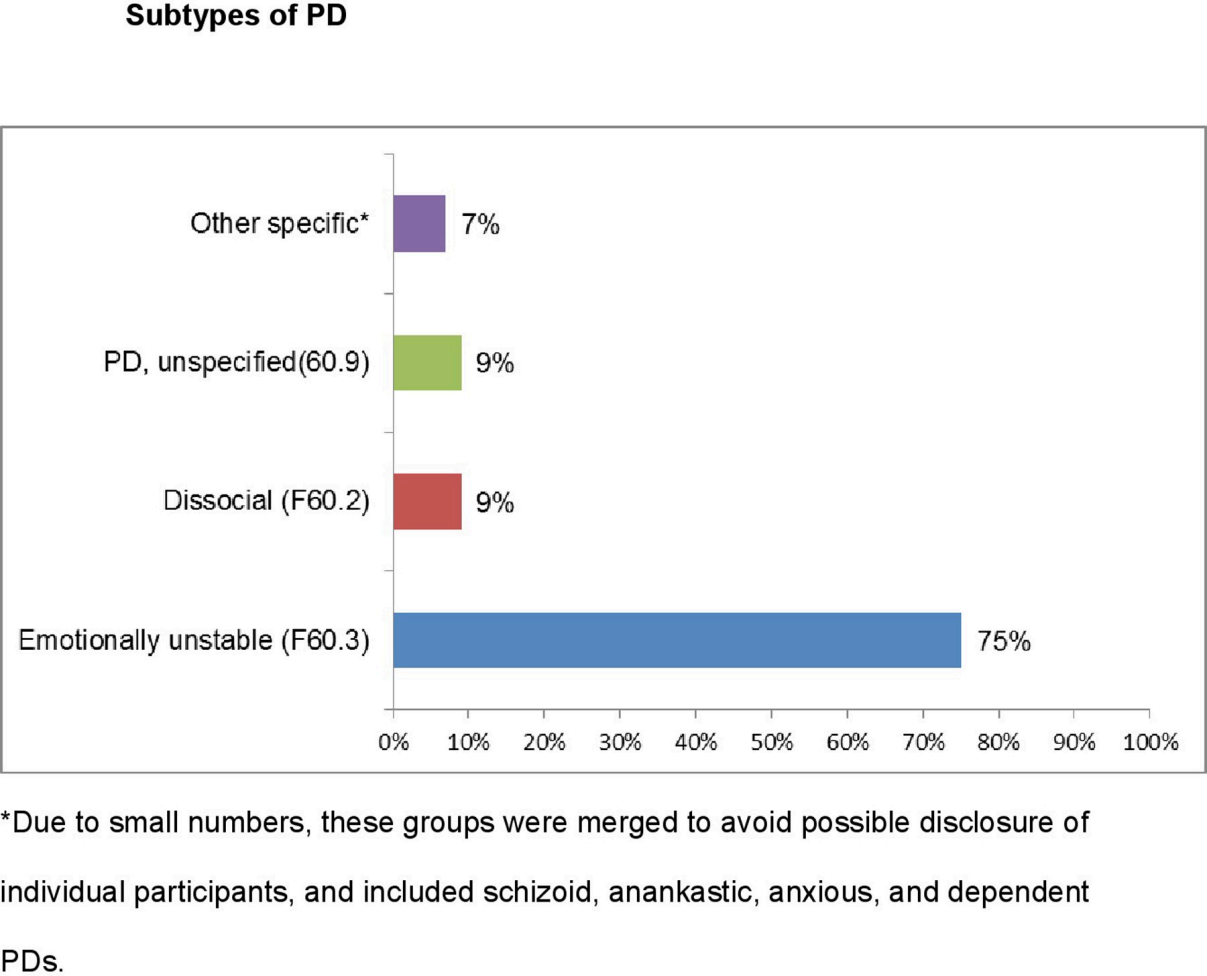
Sub-types of PD diagnosed (N = 76) among participants (ICD-10).

The majority of participants with a PD record (75%) received a diagnosis of ‘emotionally unstable’ PD (75%; see Fig 1), followed by ‘dissocial PD’ (9.2%) and ‘unspecified PD (9.2%). Due to the small sample size, we did not inspect these subtypes further in the present study.

### Missing data

Thirty-nine participants (4.9%) had missing data on either marital status or neighbourhood-factors (S1 Table). There were no significant differences between people with and without missing data, except that participants with missing data had lower individual and parental SES compared with those without missing data (both p<0.01).

### Sociodemographic and clinical features of people with PD

Univariable modelling (Table 2) confirmed the above patterns. After multivariable modelling, receiving a PD diagnosis was associated with sex, ethnicity, parental SES, FEP status, first rank delusions and hallucinations. Thus, PD was more common amongst women (odds ratio (OR): 3.36; 95% CI: 1.97–5.73), participants with lower parental SES (OR per lower category: 1.43; 95% CI: 1.12–1.84), those without comorbid FEP (OR: 2.99; 95% CI: 1.61–5.52) and those experiencing more hallucinations (OR: 1.61; 95% CI: 1.23–2.12). PD risk was weakly associated with age-at-referral (OR per year: 0.95; 95% CI: 0.89–1.00; p = 0.059), which was retained in the model. PD risk was lower in the BME group (OR: 0.27; 95% CI: 0.11–0.71), and participants with more first rank delusions symptoms (OR: 0.74; 95% CI: 0.56–0.97).

**Table 2:** Logistic regression models for predicting receiving a PD diagnosis in the EIP service.

Variable	Univariable model			Multivariable model		
	OR†	95% CI‡	AIC§	OR	95% CI	LRT¶ p-value
Age	0.92	(0.87–0.97)	467.1	0.95	(0.89–1.004)	0.059
Sex			454.9			<0.01
Male	1	-		1	-	
Female	3.18	(1.93–5.26)		3.36	(1.97–5.73)	
Ethnicity			463.1			<0.01
White British	1	-		1	-	
BME	0.23	(0.09–0.60)		0.27	(0.11–0.71)	
Marital Status			477.0			0.67
Married/Civil Partnership	1	-		1	-	
Single	1.57	(0.58–4.30)		1.26	(0.41–3.87)	
Divorced/Dissolved/Separated	2.16	(0.34–13.61)		2.62	(0.34–20.01)	
Participant SES			472.2			0.16
Professional & managerial	1	-		1	-	
Intermediate	1.13	(0.24–5.37)		0.67	(0.14–3.30)	
Routine	3.12	(0.90–10.77)		1.78	(0.50–6.32)	
Student	2.45	(0.66–9.08)		0.98	(0.24–3.99)	
LR unemployed or NW	4.05	(1.11–14.74)		1.97	(0.52–7.53)	
Parental SES			467.3			0.01
Professional & managerial	1	-		1	-	
Intermediate	3.00	(1.29–7.00)		2.68	(1.12–6.38)	
Routine	3.20	(1.42–7.19)		3.27	(1.42–7.56)	
Long-term unemployed, not working or student	3.57	(1.55–8.24)		3.44	(1.44–8.22)	
Parental SES—linear			467.1			<0.01
				1.43	(1.12–1.84)	
Country of birth			465.5			0.29
Born in UK	1	-				
Foreign born	0.16	(0.04–0.69)		0.40	(0.07–2.35)	
Having FEP			461.0			<0.01
Yes	1	-		1	-	-
No	3.39	(1.87–6.16)		2.99	(1.61–5.52)	
Waiting time (weeks)	1.04	(1.01–1.07)	469.5	1.03	(0.997–1.06)	0.08
Symptoms of Psychosis						
Mania	0.78	(0.60–1.01)	472.5	0.89	(0.66–1.21)	0.47
Depressive symptoms	1.61	(1.22–2.12)	463.6	1.25	(0.94–1.66)	0.13
Other delusions	0.88	(0.69–1.14)	475.1	0.92	(0.68–1.24)	0.59
Psychomotor poverty & disorg.	0.69	(0.53–0.89)	467.7	0.85	(0.65–1.11)	0.22
First rank delusions	0.68	(0.52–0.88)	467.2	0.74	(0.56–0.97)	0.03
Paranoia	1.26	(0.97–1.63)	473.0	1.14	(0.83–1.58)	0.42
Hallucinations	1.68	(1.28–2.20)	460.8	1.61	(1.23–2.12)	<0.01
Population Density			477.9			0.90
0–4,000 ppha	1	-		1	-	
4,001–8,000 ppha	1.36	(0.67–2.73)		1.31	(0.65–2.65)	
8,001–12,000 ppha	1.44	(0.72–2.88)		1.16	(0.58–2.32)	
12,001-max ppha	0.82	(0.34–1.99)		1.16	(0.47–2.82)	
Multiple Deprivation			476.9			0.38
7.8–18%	1	-		1	-	
18.1–28%	0.90	(0.45–1.81)		0.74	(0.36–1.49)	
28.1–38%	1.51	(0.72–3.17)		1.04	(0.50–2.20)	
38.1–47.1%	1.65	(0.55–4.98)		1.70	(0.61–4.78)	

†: Odds ratio;

‡: Confidence interval;

§: Akaike Information Criterion;

¶: Likelihood ratio test; PD: personality disorder; BME: black and minority ethnic; PPHA: people per hectare; SES: socioeconomic status

Although population density and multiple deprivation were not associated with PD in univariable models (Table 2), we re-checked these variables in our final multivariable model given our *a priori* interest in these factors; however, neither variable improved the final model (LRT_population density_ p = 0.40); (LRT_multiple deprivation_ p = 0.89).

## Discussion

### Main findings

To our knowledge, this is the first study to have investigated the prevalence and correlates of recording of PD diagnoses in a large, prospectively-collected sample in EIP services. While the overall prevalence of PD diagnoses was lower than we expected, at about 10%, PD diagnoses were more common for women and in those of white British ethnicity, as hypothesised. We also found that people with lower parental SES and those found not to meet research-based diagnostic criteria for FEP during their EIP care were more likely to receive a PD diagnosis. Contrary to our expectations, there was no evidence of an association between prevalence of PD and neighbourhood-level deprivation or population density in this sample. People reporting more hallucinations and fewer first-rank delusions, were more likely to receive PD diagnosis, underlining overlapping phenomenological presentations at first referral to EIP services.

### Meaning of findings

Our findings are partially consistent with previous studies. The prevalence of recording PD in our study was much lower compared with the 45% prevalence in a study conducted in a similar EIP setting [11], or studies carried out in secondary mental health care settings, where PD prevalence ranges from 31% to 92% [34–37]. A number of possible reasons for this may exist. For example, the present study is based on people referred to EIP services due to suspected psychosis. When psychotic symptoms become prominent enough to warrant clinical attention, the sensitivity of PD diagnosis could be weaker [3] or, alternatively, the likelihood that clinicians record a PD diagnosis may be reduced. We may have also underestimated the true prevalence of PD in our sample because, unlike previous studies [11,34–37], we did not use a structured instrument such as SCID-II to define PD. Given the naturalistic design of our cohort, we were reliant on PD diagnoses recorded during routine clinical practice. Further, while our sample was based on precise epidemiological criteria, we only assessed people accepted into EIP care; PD prevalence may be higher amongst those referred to, but not accepted by EIP services, and may indicate that these services are already effectively screening and triaging people who require onward referral to other specialist psychiatric services.

It is perhaps unsurprising that the absence of a FEP diagnosis increased the odds of receiving PD diagnosis in clinical practice, and is consistent with a small handful of people treated in EIP who, after extended evaluation, require onward referral to other specialist services. A similar result was reported in a recent study, indicating that participants at high-risk for psychosis were found to present to EIP services with more prominent personality traits and low transition to FEP at follow-up[38]. The associations we observed between PD prevalence and symptom dimensions associated with psychosis (including hallucinations and first rank delusions) is novel and underlines the phenomenological overlap and difficulties that diagnosticians may face in evaluating participants with symptoms inherent to both PD and FEP.

Participants receiving a PD diagnosis in our sample were more likely to be women, white British, and from lower SES groups, consistent with the previous literature [24,27,39,40]. While PD diagnoses were more likely to be recorded for people of white British ethnicity accepted into EIP care for suspected psychosis, our study does not provide information on the relative prevalence of PD by ethnicity in the general population. People from BME backgrounds are over-represented in FEP samples, including ours[26], so other study designs are required to determine whether the incidence or prevalence of PD varies by ethnicity.

Lastly, in contrast to our original hypothesis, we did not find associations between indicators of neighbourhood-level deprivation or population density and PD in people presenting to EIP services, primarily for psychosis. Nonetheless, we have previously shown that participants in this sample as a whole are more likely to come from more deprived and densely-populated area than the general population [26,41]. Our data suggested that people presenting to EIP services with and without a PD diagnosis tended to come from similar areas in terms of deprivation and population density, although we found strong evidence that participants who received a PD diagnosis in our sample were even more disadvantaged in terms of individual-level SES than those without PD diagnosis. Together, these findings suggest that the PD group in our sample represents an extremely socially disadvantaged group.

### Limitations

This study has some limitations. First, the original SEPEA study was designed to investigate the social epidemiology of psychotic disorders and not PD as a primary outcome; thus, as discussed above, we were reliant on PD diagnoses made during clinical practice which did not necessarily entail a structured instrument for diagnosing PD. Nonetheless, however, this may reflect the real-world assessment of PD in clinical practice in EIP services. Further, diagnosis of PD used in this analysis was made at 6 months after acceptance to EIP service or at discharge. Thus, it is not clear whether people with PD had premorbid PD before EIP or if they developed PD symptoms after acceptance to EIP.

### Conclusions

In contrast to previous studies, we did not find high levels of PD in a large, prospectively-collected cohort of people accepted into EIP services in England for suspected psychosis. This suggests that these services may be largely appropriately screening and triaging referrals to divert people with primary diagnoses of PD. Nonetheless, participants receiving PD diagnoses in our sample were less likely to receive a validated, research-based diagnosis of psychotic disorder while in EIP care, despite similarities in their symptoms. This symptomatic overlap highlights that difficulties in diagnostic assessment and categorisation, which may delay onward referral and treatment of personality-related problems. While careful assessment of PD symptoms at referral may further help to signpost people to appropriate services, these problems only become apparent during longitudinal assessment in EIP care.

In an already socioeconomically disadvantaged EIP cohort, people with PD reported more severe individual-level social disadvantage in terms of relationship status and occupational position, although we found no evidence to suggest people with PD came from even more deprived or densely populated neighbourhoods at first referral than other groups referred to EIP care for suspected psychosis.

## Supporting information

S1 Table Characteristics of the sample with and without missing data. (DOCX)

## References

[1] Lenzenweger MF, Lane MC, Loranger AW, Kessler RC. DSM-IV personality disorders in the National Comorbidity Survey Replication. Biol Psychiatry. 2007 9;62(6):553–64. 10.1016/j.biopsych.2006.09.019 17217923 PMC2044500

[2] Paris J. Why Psychiatrists are Reluctant to Diagnose: Borderline Personality Disorder. Psychiatry. 2007;4(1):35–9. 20805927 PMC2922389

[3] Newton-Howes G, Tyrer P, North B, Yang M. The prevalence of personality disorder in schizophrenia and psychotic disorders: systematic review of rates and explanatory modelling. Psychol Med. 2008;38:1075–1082. 10.1017/S0033291707002036 18070369

[4] Slotema CW, Blom JD, Niemantsverdriet MBA, Deen M, Sommer IEC. Comorbid Diagnosis of Psychotic Disorders in Borderline Personality Disorder: Prevalence and Influence on Outcome. Front Psychiatry [Internet]. 2018;9(March):1–8. Available from: http://journal.frontiersin.org/article/10.3389/fpsyt.2018.00084/full 29593589 10.3389/fpsyt.2018.00084 PMC5861147

[5] McMillan KA, Enns MW, Cox BJ, Sareen J. Comorbidity of Axis I and II mental disorders with schizophrenia and psychotic disorders: findings from the National Epidemiologic Survey on Alcohol and Related Conditions. Can J Psychiatry [Internet]. 2009;54(7):477–86. Available from: http://ovidsp.ovid.com/ovidweb.cgi?T = JS&PAGE = reference&D = med6&NEWS = N&AN = 19660170 10.1177/070674370905400709 19660170

[6] Paris J. Personality disorders begin in adolescence. J Can Acad Child Adolesc Psychiatry. 2013;22(3):195–6. 10.1007/s00787-013-0389-7 23970906 PMC3749891

[7] Kessler RC, Amminger GP, Aguilar-Gaxiola S, Alonso J, Lee S, Üstün TB. Age of onset of mental disorders: A review of recent literature. Current Opinion in Psychiatry. 2007. 10.1097/YCO.0b013e32816ebc8c PMC1925038 17551351

[8] McClellan JM, Werry JS, Ham M. A follow-up study of early onset psychosis: Comparison between outcome diagnoses of schizophrenia, mood disorders, and personality disorders. J Autism Dev Disord. 1993;23(2):243–62. 10.1007/BF01046218 8331046

[9] Stevenson J, Brodaty H, Boyce P, Byth K. Personality disorder comorbidity and outcome: Comparison of three age groups. Aust N Z J Psychiatry. 2011;45(9):771–9. 10.3109/00048674.2011.595685 21827347

[10] Moran P, Walsh E, Tyrer P, Burns T, Creed F, Fahy T. Impact of comorbid personality disorder on violence in psychosis: Report from the UK700 trial. Br J Psychiatry. 2003;182(FEB.):129–34. 12562740 10.1192/bjp.182.2.129

[11] Fornells-Ambrojo M, Pocock P, Mintah R, Barker C, Craig T, Lappin JM. Co-morbid personality disorder in early intervention psychosis clients is associated with greater key worker emotional involvement. Early Intervention in Psychiatry. 2015; 10.1111/eip.12286 26552836

[12] Simonsen E, Haahr U, Mortensen EL, Friis S, Johannessen JO, Larsen TK, et al Personality disorders in first-episode psychosis. Personal Ment Health. 2008;2:230–9.

[13] Niemantsverdriet MBA, Slotema CW, Blom JD, Franken IH, Hoek HW, Sommer IEC, et al Hallucinations in borderline personality disorder: Prevalence, characteristics and associations with comorbid symptoms and disorders. Sci Rep [Internet]. 2017;7(1):1–8. Available from: 10.1038/s41598-017-13108-6 29066713 PMC5654997

[14] Links PS, Steiner M, Milton J. Characteristics of psychosis in borderline personality disorder. Psychopathology. 1989;22(4):188–93. 10.1159/000284595 2798708

[15] Chopra HD, Beatson JA. Psychotic symptoms in borderline personality disorder. Am J Psychiatry. 1986;143(12):1605–7. 10.1176/ajp.143.12.1605 3789216

[16] Sjåstad HN, Gråwe RW, Egeland J. Affective Disorders among Patients with Borderline Personality Disorder. PLoS One. 2012;7(12). 10.1371/journal.pone.0050930 PMC3516502 23236411

[17] Marwaha S, Broome MR, Bebbington PE, Kuipers E, Freeman D. Mood instability and psychosis: Analyses of British national survey data. Schizophr Bull. 2014;40(2). 10.1093/schbul/sbt149 PMC3932088 24162517

[18] Harvey SB, Dean K, Morgan C, Walsh E, Demjaha A, Dazzan P, et al Self-harm in first-episode psychosis. Br J Psychiatry. 2008;192(3). 10.1192/bjp.bp.107.037192 18310576

[19] Large M, Babidge N, Andrews D, Storey P, Nielssen O. Major self-mutilation in the first episode of psychosis. Schizophr Bull. 2009;35(5). 10.1093/schbul/sbn040 PMC2728813 18495646

[20] Oumaya M, Friedman S, Pham A, Abou Abdallah T, Guelfi JD, Rouillon F. Borderline personality disorder, self-mutilation and suicide: Literature review. Encephale. 2008;34(5). 10.1016/j.encep.2007.10.007 19068333

[21] Baldwin P, Browne D, Scully PJ, Quinn JF, Morgan MG, Kinsella A, et al Epidemiology of first-episode psychosis: Illustrating the challenges across diagnostic boundaries through the Cavan-Monaghan study at 8 years. Schizophr Bull. 2005;31(3):624–38. 10.1093/schbul/sbi025 15944446

[22] Jackson HJ, Burgess PM. Personality disorders in the community: Results from the Australian National Survey of Mental Health and Wellbeing. Part II. Relationships between personality disorders, Axis I mental disorders and physical conditions with disability and health consultati. Soc Psychiatry Psychiatr Epidemiol. 2002;37(6):251–60. 10.1007/s001270200017 12111029

[23] Goldstein RB, Chou SP, Saha TD, Smith SM, Jung J, Zhang H, et al The Epidemiology of Antisocial Behavioral Syndromes in Adulthood: Results from the National Epidemiologic Survey on Alcohol and Related Conditions-III. 2017;78(1):90–8. 10.4088/JCP.15m10358 PMC5025322 27035627

[24] Tyrer P, Reed GM, Crawford MJ. Classification, assessment, prevalence, and effect of personality disorder. The Lancet. 2015. 10.1016/S0140-6736(14)61995-4 25706217

[25] Coid J, Yang M, Tyrer P, Roberts A, Ullrich S. Prevalence and correlates of personality disorder in Great Britain. Br J Psychiatry. 2006 5;188(5):423–31. 16648528 10.1192/bjp.188.5.423

[26] Kirkbride JB, Hameed Y, Ankireddypalli G, Ioannidis K, Crane CM, Nasir M, et al The epidemiology of first-episode psychosis in early intervention in psychosis services: Findings from the social epidemiology of psychoses in east Anglia [SEPEA] Study. Am J Psychiatry. 2017;174(2):143–53. 10.1176/appi.ajp.2016.16010103 27771972 PMC5939990

[27] McGilloway A, Hall RE, Lee T, Bhui KS. A systematic review of personality disorder, race and ethnicity: prevalence, aetiology and treatment. BMC Psychiatry [Internet]. 2010;10:33 Available from: http://ovidsp.ovid.com/ovidweb.cgi?T = JS&PAGE = reference&D = med6&NEWS = N&AN = 20459788 10.1186/1471-244X-10-33 20459788 PMC2882360

[28] Walsh Z, Shea MT, Yen S, Ansell EB, Grilo CM, McGlashan TH, et al Socioeconomic-status and mental health in a personality disorder sample: the importance of neighborhood factors. J Pers Disord [Internet]. 2013;27(6):820–31. Available from: http://ovidsp.ovid.com/ovidweb.cgi?T = JS&PAGE = reference&D = medl&NEWS = N&AN = 22984860 10.1521/pedi_2012_26_061 22984860 PMC4628287

[29] Craddock N, Asherson P, Owen MJ, Williams J, Mcguffin P, Farmer AE. Concurrent validity of the OPCRIT diagnostic system: Comparison of OPCRIT diagnoses with consensus best-estimate lifetime diagnoses. British Journal of Psychiatry. 1996. 10.1192/bjp.169.1.58 8818369

[30] McGuffin P, Farmer A, Harvey I. A polydiagnostic application of operational criteria in studies of psychotic illness: Development and reliability of the OPCRIT system. Arch Gen Psychiatry. 1991; 10.1001/archpsyc.1991.01810320088015 1883262

[31] Office for National Statistics. Standard Occupational Classification 2010. 2010;3:70.

[32] Kirkbride JB, Hameed Y, Wright L, Russell K, Knight C, Perez J, et al Waiting time variation in Early Intervention Psychosis services: longitudinal evidence from the SEPEA naturalistic cohort study. Soc Psychiatry Psychiatr Epidemiol. 2017;52(5):563–74. 10.1007/s00127-017-1343-7 28213813 PMC5423995

[33] Solmi F, Mohammadi A, Perez JA, Hameed Y, Jones PB, Kirkbride JB. Predictors of disengagement from Early Intervention in Psychosis services. Br J Psychiatry. 2018;213(2):477–83. 10.1192/bjp.2018.91 30027874 PMC6071847

[34] Beckwith H, Moran PF, Reilly J. Personality disorder prevalence in psychiatric outpatients: a systematic literature review. Personal Ment Health. 2014;8:91–101. 10.1002/pmh.1252 24431304

[35] Keown P, Holloway F, Kuipers E. The prevalence of personality disorders, psychotic disorders and affective disorders amongst the patients seen by a community mental health team in London. Soc Psychiatry Psychiatr Epidemiol. 2002;37(5):225–9. 10.1007/s00127-002-0533-z 12107714

[36] Newton-Howes G, Tyrer P, Anagnostakis K, Cooper S, Bowden-Jones O, Weaver T. The prevalence of personality disorder, its comorbidity with mental state disorders, and its clinical significance in community mental health teams. Soc Psychiatry Psychiatr Epidemiol. 2010;45(4):453–60. 10.1007/s00127-009-0084-7 19543844

[37] Ranger M, Methuan C, Rutter D, Rao B, Tyrer P. Prevalence of personality disorder in the case-load of an inner-city assertive outreach team. Psychiatr Bull. 2004;28:441–3.

[38] Llewellyn-jones JS, Camino G, Russo DA, Painter M, Montejo AL, Ochoa S, et al Clinically signi fi cant personality traits in individuals at high risk of developing psychosis. Psychiatry Res [Internet]. 2018;261(May 2017):498–503. Available from: 10.1016/j.psychres.2018.01.027 29395871

[39] Saraiva Leao T, Sundquist J, Johansson LM, Johansson S-E, Sundquist K, T.S. L, et al Incidence of Mental Disorders in Second-Generation Immigrants in Sweden: A Four-Year Cohort Study. Bain Bradby, Brindis, Cantor-Graae, Chakraborty, Elenius, Escobar, Fossion, Goodman, Harrison, Hjern, Hovey, Janssen, Johansson, Kaufman, Keskimaki, Kleinbaum, Kuusela, Lorant, Major, McKelvey, Sawyer, Sawyer, Selten, Sowa, Sundquist, Sundquist, Wiking, B, editor. Ethn Health [Internet]. 2005;10(3):243–56. Available from: http://ovidsp.ovid.com/ovidweb.cgi?T = JS&PAGE = reference&D = med5&NEWS = N&AN = 16087456 10.1080/13557850500096878 16087456

[40] Goldstein RB, Chou SP, Saha TD, Smith SM, Jung J, Zhang H, et al The epidemiology of antisocial behavioral syndromes in adulthood: Results from the National Epidemiologic Survey on Alcohol and Related Conditions-III. J Clin Psychiatry [Internet]. 2017;78(1):90–8. Available from: http://www.psychiatrist.com/JCP/article/_layouts/ppp.psych.controls/BinaryViewer.ashx?Article = /JCP/article/Pages/2017/v78n01/v78n0114.aspx&Type = Article 10.4088/JCP.15m10358 27035627 PMC5025322

[41] Richardson L, Hameed Y, Perez J, Jones PB, Kirkbride JB. Association of environment with the risk of developing psychotic disorders in rural populations: Findings from the social epidemiology of psychoses in east anglia study. JAMA Psychiatry. 2018;75(1):75–83. 10.1001/jamapsychiatry.2017.3582 29188295 PMC5833554

