# Supplementary material for: Personality disorder in an Early Intervention Psychosis cohort: Findings from the Social Epidemiology of Psychoses in East Anglia (SEPEA) study
Source: PLoS One. 2020 Jun 5;15(6):e0234047. doi: 10.1371/journal.pone.0234047 (PMC7274401; doi:10.1371/journal.pone.0234047)
Supplement: S1 Table — (DOCX) [file pone.0234047.s001.docx]

**S1 table. Characteristics of the sample with and without missing data**

|  | Without missing data  (n=759) | | With missing data  (n=39) | | statistic | |
| --- | --- | --- | --- | --- | --- | --- |
|  | n | (%) or (IQR) | n | (%) or (IQR) | *test* | p |
| Age (years) |  |  |  |  | RST | 0.67 |
| Median (IQR) | 22.4 | (19.3-27.0) | 22.1 | (19.9-24.5) |  |  |
| Sex |  |  |  |  | ChiT | 0.22 |
| Female | 267 | (35.2) | 10 | (25.6) |  |  |
| Male | 492 | (64.8) | 29 | (74.4) |  |  |
| Ethnicity |  |  |  |  | ChiT | 0.45 |
| White British | 585 | (77.1) | 28 | (71.8) |  |  |
| BME | 174 | (22.9) | 11 | (28.2) |  |  |
| Marital Status |  |  |  |  | ChiT | 0.13 |
| Married/Couple | 72 | (9.5) | 0 | (0) |  |  |
| Single | 671 | (88.4) | 31 | (100) |  |  |
| Divorced/Dissolved/Separated | 16 | (2.1) | 0 | (0) |  |  |
| Missing data |  |  | 8 |  |  |  |
| Participant SES |  |  |  |  | FET | <0.01 |
| Professional & managerial | 77 | (10.1) | 1 | (2.6) |  |  |
| Intermediate | 91 | (12.0) | 0 | (0) |  |  |
| Routine | 295 | (38.9) | 16 | (41.0) |  |  |
| Student | 164 | (21.6) | 8 | (20.5) |  |  |
| LR unemployed or NW | 132 | (17.4) | 14 | (35.9) |  |  |
| Parental SES |  |  |  |  | FET | <0.01 |
| Professional & managerial | 228 | (30.0) | 4 | (10.3) |  |  |
| Intermediate | 165 | (21.7) | 9 | (23.1) |  |  |
| Routine & manual | 207 | (27.3) | 10 | (25.6) |  |  |
| Long-term unemployed, not working or student | 159 | (21.0) | 16 | (41.0) |  |  |
| Country of birth |  |  |  |  | ChiT | 0.25 |
| Born in UK | 653 | (86.0) | 31 | (79.5) |  |  |
| Foreign born | 106 | (14.0) | 8 | (20.5) |  |  |
| First Episode Psychosis (FEP) |  |  |  |  | FET | 0.64 |
| Yes | 652 | (85.9) | 35 | (89.7) |  |  |
| No | 107 | (14.1) | 4 | (10.3) |  |  |
| Symptoms of Psychosis (median & IQR) |  |  |  |  |  |  |
| Mania | -0.16 | (-0.61-0.56) | -0.15 | (-0.45-0.43) | RST | 0.91 |
| Depressive symptoms | 0.00 | (-0.79-0.78) | -0.28 | (-1.16-0.51) | RST | 0.10 |
| Other delusions | -0.07 | (-0.64-0.73) | -0.03 | (-0.62-1.01) | RST | 0.36 |
| Psychomotor poverty & disorg. | -0.06 | (-0.60-0.58) | 0.30 | (-0.53-0.80) | RST | 0.11 |
| First rank delusions | -0.08 | (-0.56-0.56) | 0.04 | (-0.54-0.83) | RST | 0.52 |
| Paranoia | -0.04 | (-0.69-0.66) | 0.19 | (-0.73-0.87) | RST | 0.74 |
| Hallucinations | -0.05 | (-0.69-0.69) | -0.06 | (-0.70-0.69) | RST | 0.87 |
| Population Density |  |  |  |  | FET | 0.75 |
| 0-4,000 ppha | 340 | (44.8) | 4 | (50.0) |  |  |
| 4,001-8,000 ppha | 149 | (19.6) | 2 | (25.0) |  |  |
| 8,001-12,000 ppha | 153 | (20.2) | 2 | (25.0) |  |  |
| 12,001-max ppha | 117 | (15.4) | 0 | (0) |  |  |
| Missing data |  |  | 31 |  |  |  |
| Multiple Deprivation |  |  |  |  | FET | 0.44 |
| 7.8-18% | 186 | (24.5) | 2 | (25.0) |  |  |
| 18.1-28% | 336 | (44.3) | 2 | (25.0) |  |  |
| 28.1-38% | 183 | (24.1) | 3 | (37.5) |  |  |
| 38.1-47.1% | 54 | (7.1) | 1 | (12.5) |  |  |
| Missing data |  |  | 31 |  |  |  |

Abbreviations - RST: Rank-sum test; ChiT: Chi-square test; FET: Fisher’s exact test: PD; personality disorder; BME: black and minority ethnic; PPHA: people per hectare; SES: socioeconomic status
